# Robotic‐assisted burr preparation significantly improves cement penetration but not immediate fixation strength in total knee arthroplasty

**DOI:** 10.1002/ksa.12765

**Published:** 2025-07-07

**Authors:** Michael Tim‐Yun Ong, Mingqian Yu, Mingde Cao, Dennis King Hang Yee, Kevin Ki Wai Ho, Gene Chi Wai Man, Mingguang Bi, Jonathan Ng, Jiankun Xu, James Griffith, Patrick Shu‐Hang Yung

**Affiliations:** ^1^ Department of Orthopaedics and Traumatology Prince of Wales Hospital Hong Kong China; ^2^ Department of Orthopaedics and Traumatology, Faculty of Medicine The Chinese University of Hong Kong Hong Kong SAR China; ^3^ Center for Neuromusculoskeletal Restorative Medicine (CNRM) Hong Kong Science Park Hong Kong China; ^4^ Department of Orthopaedics and Traumatology Alice Ho Miu Ling Nethersole Hospital Hong Kong SAR China; ^5^ Department of Imaging and Interventional Radiology The Chinese University of Hong Kong Hong Kong SAR China

**Keywords:** cement penetration, oscillating saw, osteotomy, robotic‐assisted burr, total knee arthroplasty

## Abstract

**Purpose:**

Although robotic‐assisted total knee arthroplasty (TKA) is recognized for improving surgical precision, the effect of its bone preparation technique on cemented fixation remains unclear. This study compared the impact of robotic‐assisted burr versus oscillating saw techniques on cement penetration and fixation strength.

**Methods:**

Seven paired (*n* = 14) fresh‐frozen cadaveric specimens underwent either robotic‐assisted TKA using a burr system or conventional TKA using an oscillating saw. Computer tomography scans quantified cement penetration, while maximum failure load was determined using pull‐out testing.

**Results:**

Cement penetration was significantly greater in the burr group compared to the saw group (3.79 ± 0.84 cm³ vs. 2.67 ± 0.99 cm³, *p* = 0.03). The average pull‐out strength for the burr group has no statistical difference compared with the saw group (2939 ± 760.9 N vs. 2372 ± 760.5 N, *p* = 0.16).

**Conclusion:**

Bone preparation using a burr technique significantly improves cement penetration compared to conventional oscillating saw technique. However, this did not translate to improved immediate fixation strength in time‐zero biomechanical testing. Further clinical studies are needed to determine if enhanced cement penetration provides long‐term benefits for implant survivorship.

**Level of Evidence:**

N/A.

**Abbreviations**
BMD: bone mineral density
BMI: body mass index
CP: cement penetration
CT: computer tomography
HR‐pQCT: high‐resolution peripheral quantitative computed tomography
ROI: region of interest
SD: standard deviation
TKA: total knee arthroplasty
UKA: unicompartmental knee arthroplasty

## INTRODUCTION

Aseptic loosening after total knee arthroplasty (TKA) remains a common cause for failure and is the major cause of TKA revisions [24, 29]. A recent study found that aseptic loosening accounts for 45.1% of knee revision cases in Hong Kong, 36.8% in New Zealand, 24.0% in Sweden, and 14.0% in the Australian registry with 20 years of follow‐up, and 38.2% in the UK registry report with 15 years of follow‐up [9]. The underlying mechanisms causing instability and aseptic loosening are multifactorial, influenced by implant design, bone bed preparation and cementing techniques, inclusive of cement type, mixing time, cement thickness, application area, and temperature [15, 21, 23]. However, as a surgical procedure to resurface the damaged knee joint, the success of cemented TKA—defined by long‐term implant survival and reduced complications—depends on the stability of the implant and the biomechanical properties of the bone‐cement‐implant complex [3, 22, 32].

Previous studies have suggested the bone‐cement interface is critical for the overall fixation strength of the implant [28, 32]. Micro‐motion and subsequent bone resorption between the bone‐cement interface may lead to the presence of fibrous connective tissue membrane, which may result in a gradual loss of fixation between the bone‐cement interface over time, and eventually result in the loosening of the implant [3, 6, 23]. Improvements in cementing techniques have resulted in better implant fixation, including vacuum mixing, timing of cement application relative to viscosity, and appropriate cement restrictors [1, 23]. Aside from recent studies on optimizing cementation preparation and application techniques, proper bone bed preparation is crucial for achieving optimal cementation and ensuring long‐term implant survival [1, 28]. Several studies suggest that improving the depth of cement penetration into tibial cancellous bone may be beneficial in TKA. Ryo Sasaki et al. reported that the depth of the cement penetration directly affects the incidence of a radiolucent line >2 mm, which has been reported as a valuable sign for a preliminary diagnosis of aseptic loosening [25]. Furthermore, drilling sclerotic bone to enhance penetration has been recommended as a valid approach by expert consensus, despite the lack of clinical studies demonstrating its impact on implant survivorship [8].

While previous studies have established the importance of cement penetration for implant stability, the comparative effects of different bone bed preparation techniques on cementation quality remain poorly understood. Modern robotic‐assisted systems offer potential advantages in surface preparation precision, but few studies have directly compared these systems with traditional oscillating saw techniques using comprehensive three‐dimensional assessment methods.

The purpose of this cadaveric study is to evaluate the biomechanical characteristics of the bone‐cement interface, as well as the quality of cementation using different osteotomy tools. This study will investigate whether surface prepared by robotic‐assisted burr is a factor determining cement penetration and thus the strength of the cement‐bone interface.

## MATERIALS AND METHODS

### Cadaveric samples

**Figure 1:**
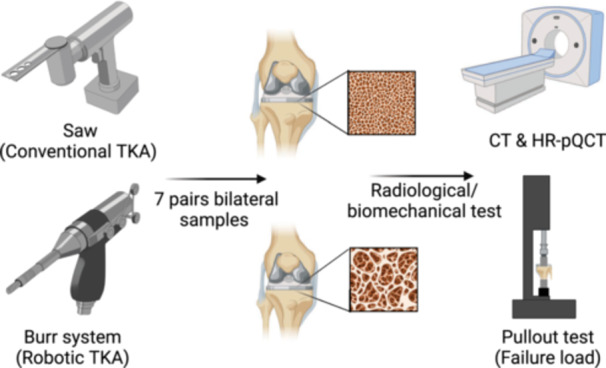
Schematic diagram of the experimental design. Seven paired knee samples were subjected to burr (robotic TKA) and saw osteotomy (conventional TKA) and cement fixation. Afterwards, CT and HR‐pQCT and biomechanical tests were performed. CT, computer tomography; HR‐pQCT, high‐resolution peripheral quantitative computed tomography; TKA, total knee arthroplasty.

The overall experimental workflow is summarized in Schematic diagram (Figure 1). Seven paired commercially available fresh‐frozen pelvis‐to‐toe cadavers were used. Prior to performing the TKA, the cadaver specimens were thawed at room temperature for 24 h. Xtreme computer tomography (CT) scans were performed for all samples to determine bone mineral density (BMD) (Scanco Medical AG). Four experienced surgeons performed the surgical procedures according to a standardized technique.

### Surgical approach

All TKAs were performed using a medial parapatellar approach. Legion (Smith and Nephew) posterior stabilized total knee replacements were used in all the cases. In each pair of bilateral cadaver knees, one side underwent TKA using the CORI burring system, while the opposite side underwent the procedure with the oscillating saw. The side allocation (right vs. left) was randomly determined to minimize bias.

### CORI burring system

In the surgeries with CORI (Smith and Nephew), the distal femoral cut was conducted with the reamer of the CORI handpiece. The handheld robotic‐assisted surgical device allows for real‐time planning and gap assessment upon a surgeon control. The cadaver was rigidly connected to CORI software via two transverse stabilization pins in the distal femur and proximal tibia. Stabilization pins, trackers and camera setup were installed to allow for navigation markers and bone movement monitors detection of the workspace within the joint. Subsequently, the remaining femoral and tibial bone cuts were performed with the CORI handpiece. Balance was achieved to within 1 mm between medial and lateral compartments in both flexion and extension.

### The standard oscillating saw technique

In the surgeries with oscillating saw, the procedure involved cutting the distal femur with an intramedullary guide and the proximal tibia with an extramedullary guide. The femur was then prepared with a four‐in‐one guide for anterior, posterior, and chamfer cuts, followed by the preparation of the box cut. Trial components were inserted, and Verasense (Orthosensor) was used to assist with soft tissue balancing. Medial and lateral compartment pressures at 10°, 45°, and 90° flexion were set to be between 5 and 40 pounds and not greater than 15 pounds difference between medial and lateral compartments.

### Cementation protocol

All the cement procedures were performed by one experienced surgeon. To cement the components, non‐antibiotic‐containing medium viscosity Rally cement (Smith & Nephew) was used. Two mixes of standard pack (40 g) were used for each knee. All cement powders and liquids were mixed at room temperature under vacuum suction to minimize porosity for 60 seconds. The bone surface was prepared with pulsatile lavage to remove loose bone, blood, fat and marrow to expose the underlying porous bone and was carefully dried afterward. The cement was manually pressurized onto the bone 180 s later. After the implants were seated, and impactor was used, the knee was held in full extension for 10 min to allow proper curing of cement. The fresh‐frozen knees were carefully dissected 20 cm from the tibia plateau. The samples were stored at −20°C in the deep freezer until further experiment.

### Radiological cement penetration analyses

Tibia plateau bone cement penetration was evaluated by CT scans using the standard protocol for all the samples before the pull‐out test (Siemens Healthcare SOMATOM Drive Dual Source CT; spatial resolution 0.3 mm) based on the previously described methods [27, 28]. The cement was segmented from the CT scans and analysed using 3D slicer [14] software (http://www.slicer.org). The threshold for penetrated cement was set to 800~1300 for ROI contour. Anomalous signals were manually trimmed or removed. The volume of the penetrated cement was measured using the “segment statistics” module. For the depth of infiltration, the NIFTI file of cement segmentation was exported to ImageJ (1.52k) to generate a top‐view “z‐project” imaging. The quantitative analysis was performed using the “Plot z‐axis profile.” Pseudo‐colouring of infiltration depths generated from Z‐stacks sum slides data.

### Measurement of BMD

BMD was measured by high‐resolution peripheral quantitative computed tomography (HR‐pQCT) scanner, XtremeCT (Scanco Medical AG) and calculated by the built‐in software, expressed in milligrams hydroxyapatite per cubic centimetre (mg HA/cm³) (scanco.ch/analysis.html).

### Biomechanical test

The samples were thawed at room temperature for 12 h before the pull‐out test. Maximum failure load was determined using a pull‐out test with a cross‐head speed increment of the material testing machine (Hounsfield H25K‐S). Implant extractor (Smith & Nephew) was connected to the testing machine and was used to clamp the sample. The samples were fixed in custom‐built bottom molds. The remaining space between the specimen and mold was filled with synthetic resin (AB two‐component Epoxy resin, Qiangnai Beijing) for further fixation. Traction forces were increased constantly (5 mm/min) until implant‐cement‐bone anchoring failed. The maximum failure load was determined in the axial direction of the prosthesis stem as previous reported [11, 18].

### Visual inspection and image analysis

Upon completion of the pull‐out test, the cement adhesion to the undersurface of implants was visually inspected and photographed. The location of failure was classified into three categories—Implant‐cement interface failures, bone failures, and mixed failures based on what remained adherent to the undersurface of the tibial tray as previously reported [19].

### Data analysis

Descriptive statistical methods were used in the present pre‐clinical investigation to summarize the pull‐out force. Frequency counts and percentages were used to summarize failure locations. A Wilcoxon matched pairs test was used to test for differences in pull‐out force and bone cement penetration between the two groups. Fisher's exact test was employed to examine differences in failure patterns. Continuous parameters are presented with their means, standard deviations (SDs). All statistical analyses were completed using SPSS statistical software, version 22.0 and visualized by GraphPad Prism, (Version 10.3.0 (461)). *p* < 0.05 were considered statistically significant.

## RESULTS

The cadaver donor demographic data are shown in Table 1. There were three male donors and four female donors, with the age ranging from 57~77 years old.

**Table 1:** Demographic data.

No.	Age (years)	Height (cm)	Weight (kg)	BMI (kg/m²)	Gender	Race
1	63	160.00	51.71	20.19	F	Caucasian
2	66	162.56	73.94	27.98	F	Caucasian
3	57	165.10	60.33	22.13	F	African American
4	68	177.80	48.08	15.21	M	Caucasian
5	69	182.88	68.04	20.34	M	Caucasian
6	77	172.72	49.90	16.72	M	Caucasian
7	68	165.16	90.72	33.32	F	Caucasian

**Figure 2:**
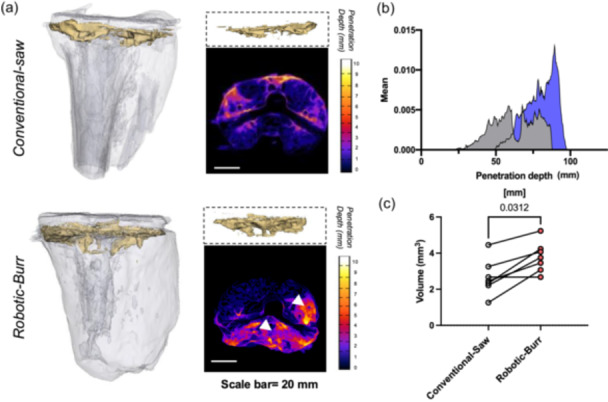
Robotic‐burr methods has better cement penetration. (a) Representative 3D reconstruction of TKA tibia and the cement mantle; Heat map of cement vertical infiltration. Scale bar = 20 mm; white arrows indicate areas of deeper infiltration. (b) Representative distribution of cement penetration depth of the tibial plateau of two groups. (c) The comparison of the cement penetration volume of the two group, *n* = 7, paired Wilcoxon test.

Cement penetration was significantly greater in the burr group compared to the saw group (3.79 ± 0.84 cm³ vs. 2.67 ± 0.99 cm³, *p* = 0.03) (Figure 2). The heat map of cement vertical infiltration generated by Z‐stack demonstrated the depth of cement under the bone surface. The brighter colour indicated better cement infiltration into the bone in the saw group (Figure 2).

**Figure 3:**
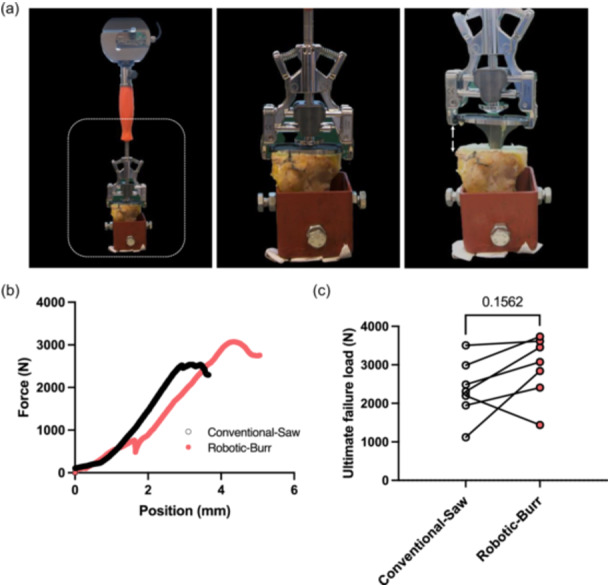
Biomechanical test reveals no significant difference in terms of the ultimate failure load. (a) Pull‐out test set up with the material testing machine (Hounsfield H25K‐S). (b) Representative Force/position curve of the pull‐out test. (c) The comparison of the cement penetration volume of the two groups, *n* = 7, paired Wilcoxon test.

No statistically significant difference was found in mean BMD between burr group (743.27 ± 61.23 mg HA/ccm) and saw group (723.30 ± 49.31 mg HA/ccm) (*p* = 0.30). The mean cortical BMD for all specimens is 885.1 ± 71.78 mg HA/ccm, and the mean trabecular BMD is 175.6 ± 55.83 mg HA/ccm. Notably, there are no significant differences between the paired groups (Supporting Information S1: Figure S1). Additionally, there was no statistical difference in the average pull‐out strength for the burr group compared with the saw group (2939 ± 760.9 N vs. 2372 ± 760.5 N, *p* = 0.16) (Figure 3).

**Figure 4:**
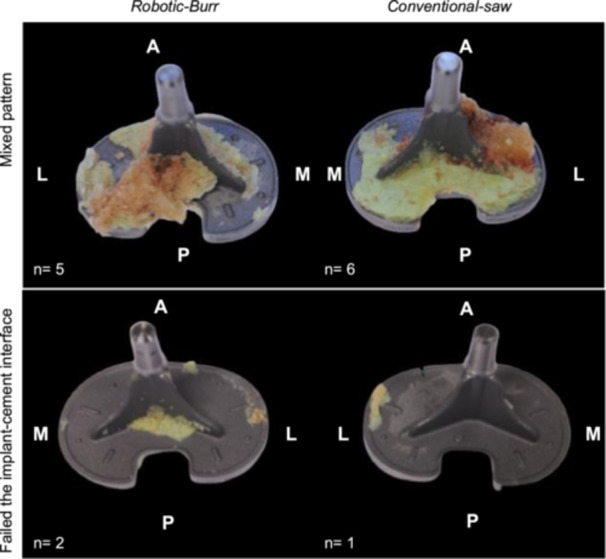
Representative explant images across different failure patterns. (Upper panel) Mixed pattern of failure. Specimen failed at the bone‐cement interface, with adjacent areas of failure of the implant‐cement interface. (Lower panel) Samples failed solely at the implant‐cement interface. A, anterior side; L, lateral side; M, medial side; P, posterior side.

Two failure patterns were identified after the pull‐out test. Most of the specimens failed at the bone‐cement interface, with adjacent areas of failure of the implant‐cement interface implant cement interface (11/14), representing a mixed pattern of failure. In contrast, only a small portion of samples failed solely at the implant‐cement interface (3/14) (Figure 4). There is no group difference in terms of the failure patterns.

## DISCUSSION

The primary finding of the present study was that bone preparation with robotic‐assisted burr systems significantly improves cement penetration compared to conventional oscillating saw techniques, although this did not translate to statistically significant differences in immediate fixation strength.

Previous studies have demonstrated that a variety of technical factors in TKA could potentially affect the penetration of cement and thus long‐term outcomes, such as tourniquet use [30], mixing under vacuum conditions [4], pulsatile lavage [7], negative pressure intrusion cementing technique [2], and motion during Cementing [19]. However, few studies have demonstrated the effect of different osteotomy techniques on cement infiltration and bone bed preparation.

Our results demonstrated for the first time that preparation with a robotic‐assisted burr system resulted in greater cement penetration volume compared to conventional oscillating saw techniques, although the difference in pull‐out strength did not reach statistical significance.

The cement penetration volumes observed in the burr group (3.79 ± 0.84 cm³) were significantly higher than in the saw group (2.67 ± 0.99 cm³, *p* = 0.03), suggesting improved interdigitation. Scheele et al. noted that bone cement achieves fixation through intrusion into trabecular bone microstructure and mechanical interlocking rather than adhesion or superficial conformity with the bone bed [26]. The primary stability of cemented fixation depends on cement penetration into adjacent trabecular bone, which serves as an indicator of biomechanical stability [26].

The mechanisms underlying the improved cement penetration likely relate to several factors. First, oscillating saws are known to produce a relatively smooth cut surface (Flatness 0.15–0.40 mm) with a layer of debris that can potentially interfere with cement interdigitation [31]. In contrast, burring systems may create a more textured surface with exposed trabecular channels that facilitate cement flow. Second, the precision of robotic‐assisted burring systems may allow for more consistent bone preparation, minimizing areas of inadequate surface treatment, especially at the medial tibia plateau.

The lack of statistical significance in the pull‐out strength (2939 ± 760.9 N vs. 2372 ± 760.5 N, *p* = 0.16) may be attributed to several factors. The time‐zero fixation strength may not fully capture the long‐term biomechanical advantages of improved cement penetration. Initial fixation may be more dependent on other factors such as implant design, cement technique, or bone quality, while the benefits of enhanced cement penetration might become more apparent over time through improved resistance to micromotion and better long‐term integration.

In terms of the failure modes, we did not observe any cases of complete failure of the bone. Instead, we identified two types of failure: mixed mode (11/14, 78.6%) and failure of fixation at the implant‐cement interface (3/14, 21.4%). Previous study has suggested that failure of fixation at the implant‐cement interface should be the predominant failure pattern [19]. This discrepancy could be attributed to differences in the pullout devices used. In our study, we employed a commercial extractor instead of a custom‐designed pullout device, which might have introduced eccentric loading. Additionally, variations in prosthesis design could also contribute to these differences [13]. Overall, there was no significant difference in failure patterns between the two groups. Focusing on the most common mixed failure pattern, the robotic‐burr group exhibited more pronounced cement penetration in the posterior and lateral plateau regions, as revealed by the CT penetration analysis (Figure 2). Furthermore, the robotic‐burr group showed more bone‐cement composite attached to the implant in the lateral and posterior regions (Figure 4). This may indicate that the robotic‐burr group achieved better cement penetration in the posterolateral regions.

Previous studies have shown that the cement‐bone interface is critical for total knee replacement outcomes [7, 11]. The current study evaluated and compared the biomechanical characteristics and the cementation quality of two different bone bed preparation techniques—one by robotic burr and one by oscillating saw. While the results demonstrated significantly improved cement penetration with robotic burr preparation, this did not translate to statistically significant differences in immediate fixation strength.

There is mixed evidence for robotic versus conventional TKA. Hoveidaei AH et al. found no difference in patient satisfaction outcomes in the short to mid‐term for robotic TKA compared to conventional methods [10]. However, a nationwide database study of robotics for TKA found lower revision rates, lower incidences of manipulation under anaesthesia, decreased occurrence of systemic complications, and lower opiate consumption for postoperative pain management compared to conventional counterparts [20]. Our results suggest that one of the potential overlooked advantages of robotic total knee arthroplasty (TKA) could be the preparation of the bone bed using a burr system. It is worth noting that not all robotic systems adopt the burr system. While others, such as ROSA and NAVIO, are also using the saw‐controlled jig system [18]. A side‐by‐side comparison of these approaches may help us to increase our understanding of using a burr to prepare the bone bed independently of accuracy improvement. Notably, the Mako system for robotic Unicompartmental Knee Arthroplasty (UKA) also employs a robotic‐arm with burr system for osteotomy. Alisdair Gilmour et al. reported a randomized controlled trial comparing Robotic‐Arm‐Assisted versus Conventional UKA, examining 2‐year clinical outcomes, which demonstrated 100% survivorship in the robotic‐arm‐assisted group compared to 96.3% in the conventional group [5]. This improved survivorship may be partially attributed to the benefits of enhanced initial cement penetration provided by the robotic burr system. Given that large registry studies report higher rates of aseptic loosening in UKA compared to TKA [17], future research should investigate whether utilizing robotic‐burr systems contributes to reducing long‐term aseptic loosening rates in UKA.

Another concern when applying the burr system is about thermal necrosis. Gwenllian Tawy et al. conducted a study comparing thermal generation between burring and sawing with and without irrigation in TKA. Their findings showed comparable surface temperatures when procedures were performed without irrigation or when pre‐cooled 4° saline was used. However, surface temperatures were significantly higher in the burr group when room temperature saline was used for irrigation. Although factors such as Shannon burr geometry, rotational speed, and material type may influence heat generation, thermal necrosis remains a significant risk factor that cannot be overlooked. In the present study, irrigation was not used in either group, meaning thermal generation and related thermal necrosis would not affect the time‐zero comparison between groups. In clinical practice, a pulsed burring technique combined with pre‐cooled saline irrigation is recommended to prevent collateral thermal necrosis and maximize the advantages of the burring procedure.

Another important consideration is the potential impact of using fresh frozen cadavers on both imaging and biomechanical testing in this study. Bryan Kaye et al. reported that freezing and thawing processes result in degradation of the mechanical properties of the tibia, which was consistently found to be 15% or less [12]. Lee et al. reported that freezing does not significantly alter trabecular bone properties [16]. This suggests that cement penetration patterns in our specimens likely resemble those that would occur in clinical patients, validating the accuracy of our imaging assessments.

Several limitations must be acknowledged in this study. First, our restricted sample size (*n* = 7) limits statistical power and may have prevented detection of significant differences in pull‐out strength despite the observed trend. Second, our pull‐out testing represents a simplified loading scenario compared to the complex forces experienced in vivo and only measures time‐zero fixation strength. The clinical benefits of improved cement penetration may manifest over time through better resistance to micromotion and wear debris, which our acute testing cannot capture. Third, cadaveric models cannot account for biological healing responses and remodelling that occur at the bone‐cement interface over time. Additionally, our cadaveric specimens did not necessarily have end‐stage osteoarthritis with the typical sclerotic medial plateau seen in clinical patients. The bone quality differences between our specimens and actual TKA candidates could influence cement penetration patterns and fixation strength. Finally, the surgeries were performed under ideal laboratory conditions, which may not fully replicate the challenges of the clinical environment, including blood, marrow extravasation, and time constraints. These factors could potentially affect cement penetration and fixation quality in real‐world settings.

## CONCLUSION

This study demonstrates that robotic‐assisted burr systems significantly enhance bone cement penetration compared to conventional oscillating saws, though immediate fixation strength remains comparable. Improved cement interdigitation may enhance long‐term implant survival by resisting micromotion and wear debris, highlighting potential clinical benefits for TKA. Future long‐term studies are needed to confirm these findings’ impact on clinical outcomes and revision rates.

## AUTHOR CONTRIBUTIONS

*Concept and design*: Michael Tim‐Yun Ong, Mingqian Yu. *Surgical procedure*: Michael Tim‐Yun Ong, Kevin Ki Wai Ho, Dennis King Hang Yee and Jonathan Ng. *Acquisition, analysis, or interpretation of data*: Mingqian Yu, Mingde Cao and Mingguang Bi. *Drafting of the manuscript*: Mingde Cao and Mingqian Yu. *Critical review of the manuscript for important intellectual content*: Jiankun Xu and James Griffith. *Statistical analysis*: Mingde Cao. *Supervision*: Michael Tim‐Yun Ong and Patrick Shu‐Hang Yung. All authors had full access to all the data in the study and read and approved the final version of the manuscript.

## CONFLICT OF INTEREST STATEMENT

The authors declare no conflicts of interest.

## ETHICS STATEMENT

This study complied with the Declaration of Helsinki after obtaining approval from the Joint Chinese University of Hong Kong‐New Territories East Cluster Clinical Research Ethics Committee (CUHK‐NTEC CREC, approval number: 2023.148). The specimens used in this study were obtained from Science Care, a whole‐body donation organization. Donors or their legal representatives provided informed consent for the use of their remains for scientific research purposes prior to donation.

## Supporting information

Rev1 Supplement 1.pdf.

## Data Availability

The data sets generated and/or analysed during the current study are available from the corresponding author upon reasonable request.
