# Supplementary figures and images for: Robotic‐assisted burr preparation significantly improves cement penetration but not immediate fixation strength in total knee arthroplasty
Source: Knee Surg Sports Traumatol Arthrosc. 2025 Jul 7;33(11):3935–43. doi: 10.1002/ksa.12765 (PMC12582227; doi:10.1002/ksa.12765)

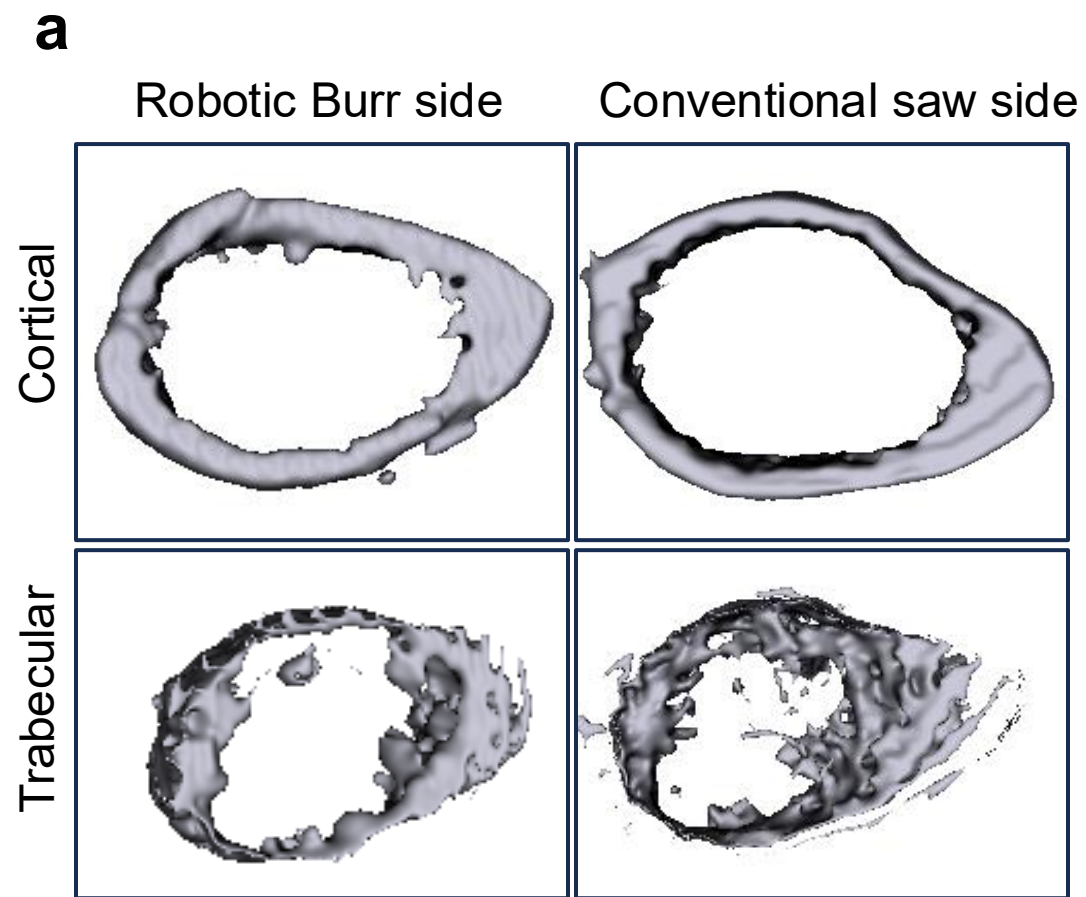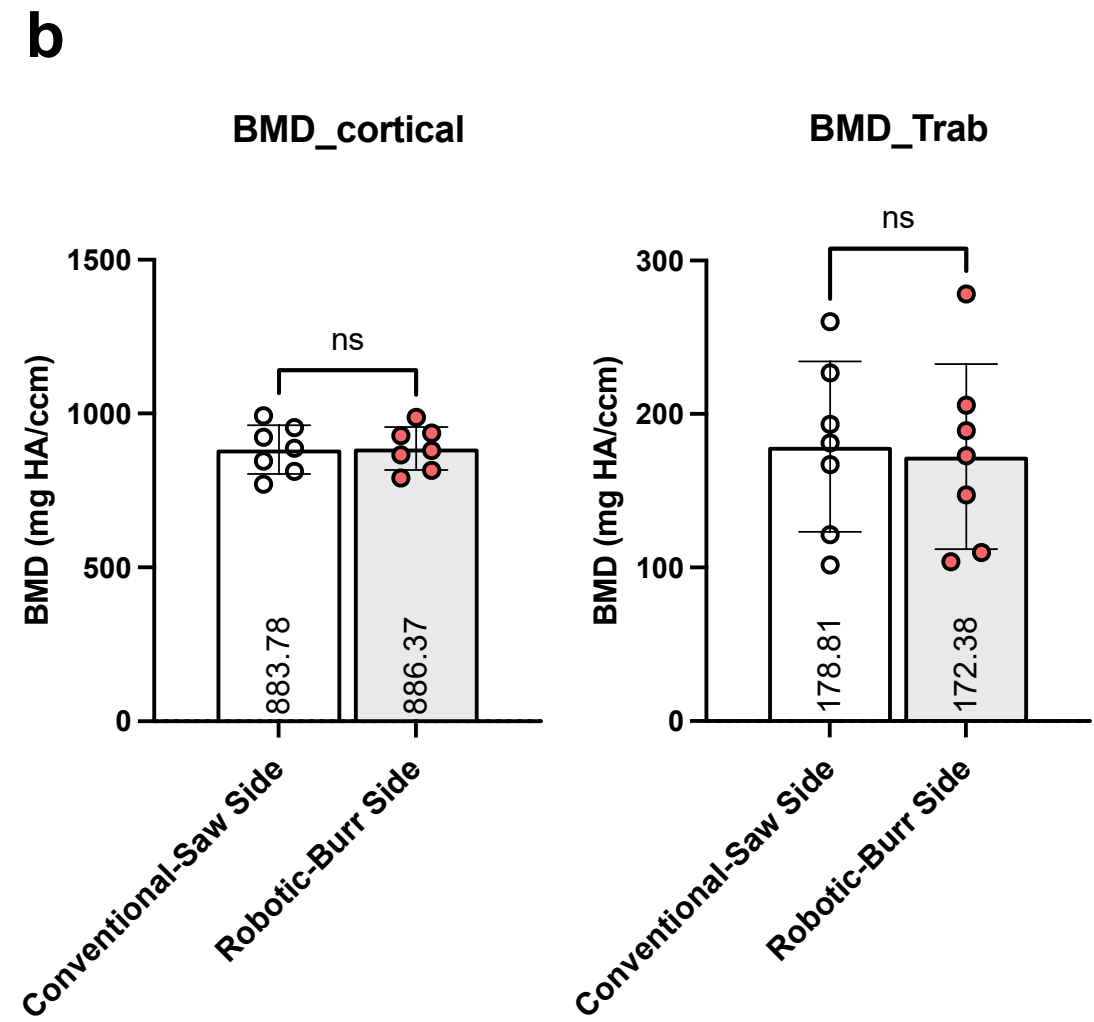

Supplement: Supplementary file 1 — Rev1 Supplement 1.pdf. [file KSA-33-3935-s001.pdf]
